# Investigating subregional PD-L1 expression within primary tumors to predict clinical outcomes in advanced NSCLC patients who received ICB-based therapy

**DOI:** 10.3389/fonc.2025.1497279

**Published:** 2025-10-17

**Authors:** Danhong Zhou, Ziwen Zhu, Jingyu Mao, Meiqin Su, Cheng Chen

**Affiliations:** Department of Respiratory and Critical Medicine, The First Affiliated Hospital of Soochow University, Suzhou, China

**Keywords:** PD-L1, immunotherapy, heterogeneity, predictive biomarker, tumor response

## Abstract

**Background:**

Programmed cell death-ligand 1 (PD-L1) immunohistochemical expression currently is the only approved useful biomarker associated with the PD-1/PD-L1 immune checkpoint blockade (ICB) efficacy for non-small cell lung carcinoma (NSCLC) patients. However, different tumor biopsy strategies could reflect the substantial heterogeneity of PD-L1 within the same tumor (spatial heterogeneity). Therefore, we aimed to explore the impact of spatial heterogeneity on the predictive value of PD-L1 expression in NSCLC patients on the ICB treatment after two cycles.

**Methods:**

All consecutive subjects with NSCLC receiving first-line ICB-based therapy for at least two cycles between January 2020 and March 2024 were enrolled and classified according to the biopsy strategies. Transbronchial lung biopsy (TBLB) or transbronchial mucosal biopsy was performed to obtain samples from the primary tumor superficial (PT_sup_) region. Moreover, endobronchial ultrasound-transbronchial needle aspiration (EBUS-TBNA) or percutaneous cutting needle biopsy (PCNB) was performed to get the primary tumor deep region (PT_deep_). The predictive capacity of PD-L1 TPS to ORR from these two sites was assessed and compared by logistic regression analysis and ROC curve analysis. The prognostic value of PT_deep_- and PT_sup_-related PD-L1 TPS to PFS was also expanded by performing propensity score matching as well as stratified analysis.

**Results:**

Among NSCLC receiving ICB therapy, PT_sup_-related PD-L1 TPS ≥50% was not associated with higher ORR (15.8% vs. 26.1%, P = 0.197) by stratified analysis. Instead, PT_deep_-related PD-L1 TPS ≥50% could bring substantially a higher ORR than those with TPS <50% (52.4% vs. 17.4%, *P* = 0.025). Furthermore, cross analysis displayed that the PD-L1 TPS <50% from the superficial or deep subregion reached relatively similar ORRs (15.8% vs. 17.4%, P = 0.861), whereas patients with PTdeep-related PD-L1 TPS ≥50% manifested a higher ORR than those with PTsup TPS ≥50% (52.4% vs. 26.1%, P = 0.036). Moreover, PT_deep_-related PD-L1 yielded the best performance in area under the curve (AUC) to predict the ORR (AUC = 0.699, *P* = 0.032) than random PD-L1 TPS (AUC=0.627, P=0.022) and PT_sup_-related PD-L1 TPS (AUC = 0.589, *P* = 0.204). As for the PFS, patients with PT_deep_-related PD-L1 TPS ≥50% had a significantly superior PFS (mPFS 19.4 vs. 10.8 months; P = 0.006) compared with patients with PT_deep_-related PD-L1 TPS <50%. After conducting matched and stratified analysis to control for potential confounding factors including immunotherapy agents and gender, PT_deep_-related remained the most stable predictor for PFS.

**Conclusions:**

PD-L1 from the deep subregion is a more solid predictive biomarker of both short- and long-time efficacies of ICB-based therapy, and optimizing the assessment of spatial heterogeneity provides a new perspective for clinicians to screen advanced NSCLC patients who can benefit from ICB-based therapy.

## 1 Introduction

Although there are many treatment options for non-small cell lung carcinoma (NSCLC) such as radiotherapy, platinum-based chemotherapy, and targeted molecular therapy, the efficacy seems to have reached a plateau and the 5-year survival rate of NSCLC patients is less than 18% (1, 2). The emergence of PD-1/PD-L1 immune checkpoint blockade (ICB) targeting the programmed cell death-1 (PD-1) and the programmed cell death-ligand 1 (PD-L1) pathway has revolutionized treatments for NSCLC. Combined PD-1/PD-L1 inhibitor monotherapy based on platinum-based chemotherapy demonstrates a relatively higher response rate, a prolonged survival time, and a favorable adverse event profile in the treatment of advanced NSCLC according to several studies (3, 4).

Since the response rates to ICB are quite low for advanced NSCLC in unselected subjects to some degree (5, 6), substantial effort is necessary to be invested in finding mechanism-based predictive biomarkers to identify the patients who will respond best to PD-1/PD-L1 pathway inhibition. PD-L1 immunohistochemical expression currently is the only approved useful biomarker associated with the ICB efficacy for NSCLC (7, 8). Recent studies have suggested that high PD-L1 immunohistochemical expression derives superior benefit from ICB therapy (9, 10). Yet, most studies have revealed the presence of a relatively modest response in patients with negative and low PD-L1 expression, which argued against the value of PD-L1 as an exclusionary predictive biomarker (8).

It is a remarkable fact that the complex interaction between tumor cells, immune cells, and their longitudinal temporal evolution can lead to spatial heterogeneity (11). One of the explanations for the limited predictive role of tumor PD-L1 expression is the spatial heterogeneity of PD-L1 expression. PD-L1 expression levels tend to vary substantially across different anatomic sites. An original study has reported that PD-L1 expression in the lung primary lesion and distant metastases had an 82% discordance rate (12). Another study has found that the combined positivity score category of PD-L1 was inconsistent when comparing different areas within the same excised head and neck squamous cell carcinoma tumor (13). Moreover, different histopathological scenarios, including heterogeneity of different subregional PD-L1 expressions of the primary tumor, are a well-documented phenomenon. All of the above may compromise the predictive power of PD-L1, and the current literature in this regard is inadequate, precluding firm conclusions.

Thus, we decided to conduct a retrospective study with the primary objective to assess and compare the prediction capacity of superficial or deep subregional PD-L1 expression of primary tumor in the NSCLC for ICB-based therapy efficacy and provide a reference for clinicians to choose the better biopsy site so that subjects suitable for ICB can be selected more accurately.

## 2 Methods

### 2.1 Study population

**Figure 1:**
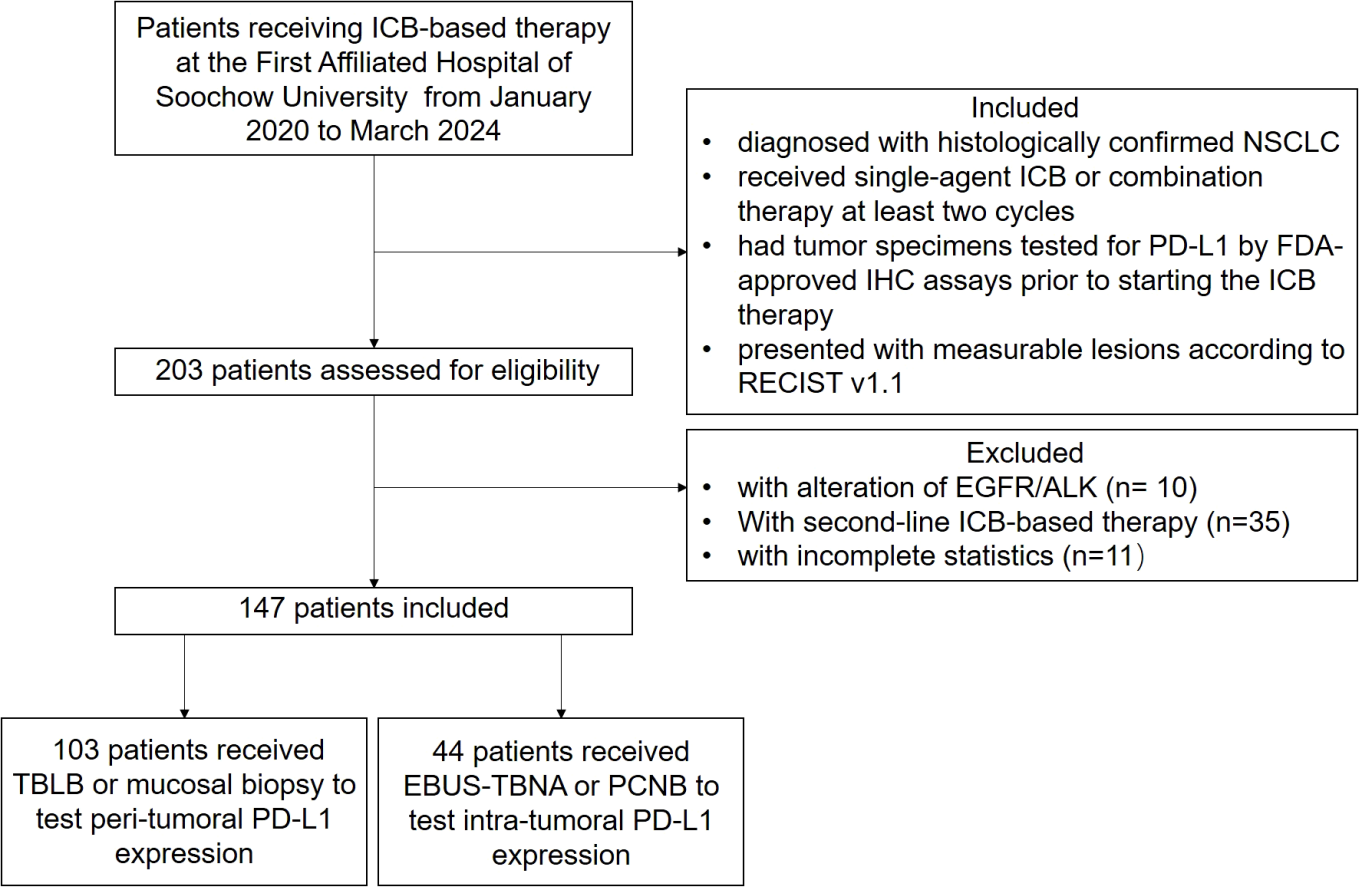
Flowchart of the study. A total of 147 patients were enrolled in the study and received at least two cycles of first-line PD-1/PD-L1 blockade immunotherapy.

We enrolled retrospectively 147 subjects with NSCLC receiving first-line ICB-based therapy at one tertiary hospital from January 2020 to March 2024. Enrolled subjects satisfied the following eligibility criteria: (1) diagnosed with histologically confirmed NSCLC without alteration of EGFR/ALK, (2) received first-line single-agent ICB or combination therapy for at least two cycles, (3) had tumor specimens tested for PD-L1 by FDA-approved immunohistochemistry (IHC) assays prior to starting the ICB therapy, and (4) presented with measurable lesions according to Response Evaluation Criteria in Solid Tumors (RECIST) v1.1 (**Figure 1**). Furthermore, patients or the public were not involved in the design, or conduct, or reporting, or dissemination plans of our research.

### 2.2 Data collection

Demographic and clinicopathological information were all derived from the electric database of the hospital. The baseline data such as age, gender, and smoke history were retrospectively collected. Information concerning disease characteristics such as PD-L1 expression level, pathological type and tumor size, and combined therapy such as chemotherapy and anti-angiogenic therapy was also obtained.

### 2.3 Clinical definition

According to NCCN Guidelines Version 7.2025 Non-Small Cell Lung Cancer, monitoring is recommended during initial therapy with response assessment with CT, with or without contrast, of known or high-risk sites of disease after two cycles and then every two to four cycles (14). This evaluation is critical for determining the continuation and potential modification of subsequent treatment strategies. Meanwhile, the selection of a two-cycle evaluation is intended to minimize the potential influence of concomitant therapies, such as radiotherapy. Thus, tumor assessments were performed based on computed tomography imaging evaluation as defined by RECIST 1.1 at baseline and two cycles of ICB-based therapy thereafter. Tumor response to ICB therapy was classified into complete response (CR), partial response (PR), stable disease (SD), and progressive disease (PD). Moreover, the objective response rate (ORR) was obtained by combining the proportions of patients achieving CR or PR as a percentage of the number of patients treated. Furthermore, disease control rate (DCR) was defined as the sum of the percentage of patients achieving CR, PR, and SD. Considering that the short evaluation window may misclassify treatment benefit, progression-free survival (PFS) was selected as a long-term endpoint to further validate the data.

### 2.4 PD-L1 testing

PD-L1 testing of 147 subjects was performed prior to starting ICB or any relevant chemotherapy and anti-angiogenic therapy. In brief, PD-L1 expression was detected by the 22C3 antibody, quantified by tumor proportion score (TPS) and defined as negative or low (0%-49%) and high (≥50%) (9, 15). Moreover, regardless of the biopsy modality, the PD-L1 expression in the entire population was defined as random PD-L1 expression.

### 2.5 Interventional lung biopsy

**Figure 2:**
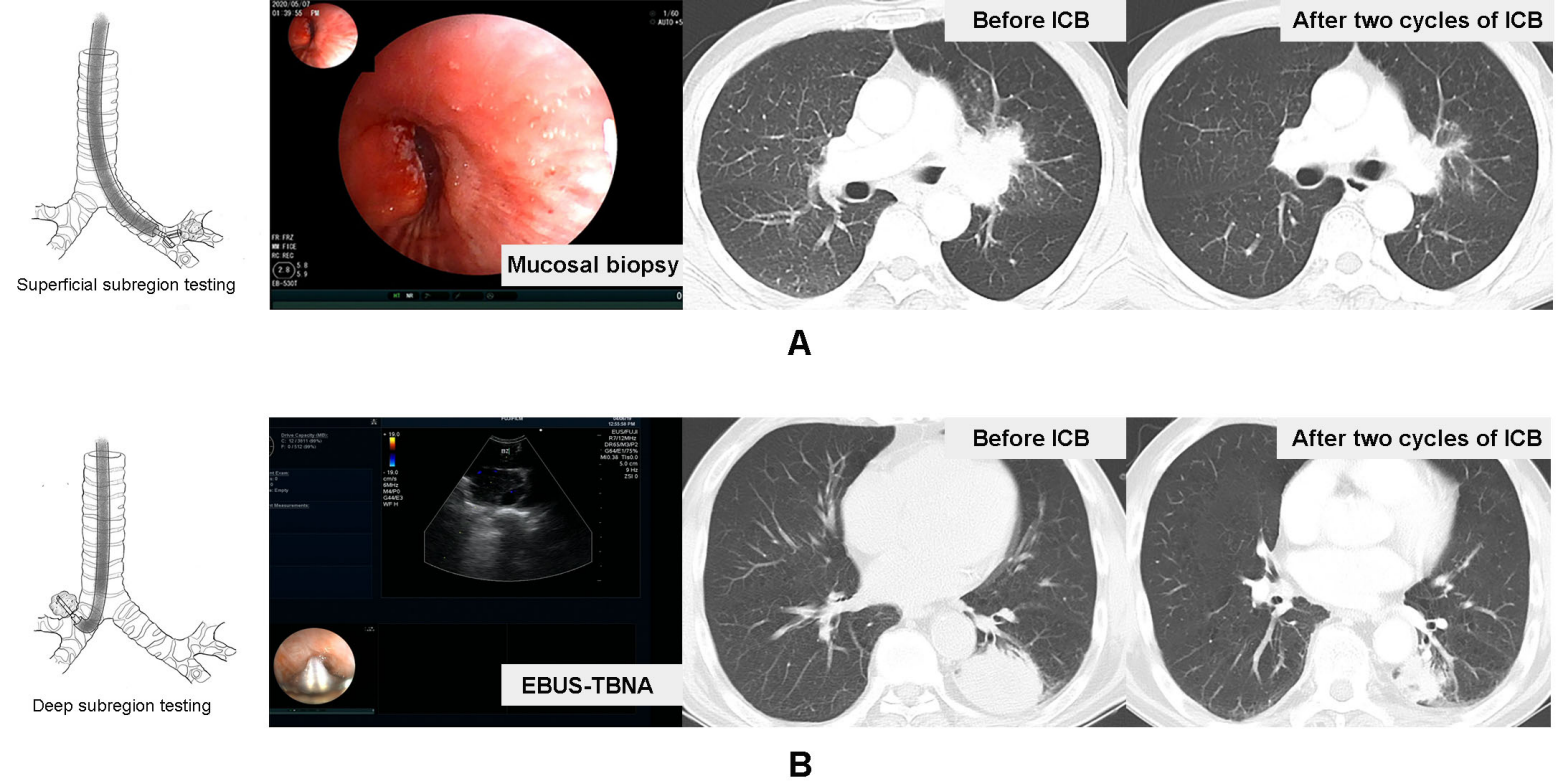
Representative patients in the tumor surface-sampled cohort and intratumor-sampled cohort. A case received mucosal biopsy of left principal bronchus to test primary tumor superficial subregion-related PD-L1 expression, and the therapeutic effect of the two-cycle ICB-based therapy was the ORR **(A)**. Another case received EBUS-TBNA to lesion in the inferior lobe of the left lung to test the deep subregion-related PD-L1 expression, and the therapeutic effect of two-cycle ICB-based therapy was also the ORR **(B)**.

In our study, we mainly utilized endobronchial ultrasound-transbronchial needle aspiration (EBUS-TBNA) to tumor, image-guided percutaneous cutting needle biopsy (PCNB), transbronchial lung biopsy (TBLB), and transbronchial mucosal biopsy to collect sufficient samples for diagnosis, histological subtyping, and PD-L1 testing. According to the principle and basic procedures of these biopsy modalities (16–18), all subjects were further divided into primary tumor superficial (PT_sup_) subregion-sampled cohort (TBLB, transbronchial mucosal biopsy) and primary tumor deep (PT_deep_) subregion-sampled cohort (EBUS-TBNA, PCNB). Based on the accessibility of the biopsy forceps and puncture needle, the subjects were divided into two groups. The biopsy forceps could reach a depth of 2 mm, which did not extend to the center of the tumor, thus constituting the PT_sup_ cohort. In contrast, the puncture needle could access the center of the tumor, thereby defining the PT_deep_ cohort. Moreover, the representative patients in these two cohorts are shown in **Figure 2**.

### 2.6 Statistical analysis

Statistical analyses were performed using the SPSS 26.0 software program. Continuous non-normal distribution variables are expressed as median (interquartile range). Categorical variables were presented as the frequency (n) and percentage (%). For group comparisons, Fisher’s exact test or chi-square test was applied for categorical variables. Uni- and multivariate non-conditional logistic regression models were used to determine the variables associated with the ORR of ICB-based therapy, and forest maps were performed accordingly. To assess and compare the accuracy of PD-L1 from different biopsy sites of the primary lung lesion as continuous variables, receiver operating characteristics (ROC) curves and waterfall plots were performed by the GraphPad Prism 6.0 software program. Kaplan–Meier survival analysis was used for analyzing the association between different subregional PD-L1 TPS and PFS. Moreover, propensity score matching and stratified analysis were applied to minimize the potential impact of confounding factors. Statistical significance was set at P < 0.05.

## 3 Results

### 3.1 Demographic and clinical characteristics of all subjects

A total of 147 NSCLC subjects who received first-line ICB-based therapy for at least two cycles at one tertiary hospital from January 2020 to March 2024 were included. The demographic and clinicopathologic characteristics of these subjects are summarized in **Table 1**. The median age of overall subjects was 68 (range 63.0, 73.0) years; the majority of subjects were men (n=133, 90.5%). 63.3% of the subjects were current or former smokers. Moreover, squamous cell carcinoma was the most common histology (n=88, 59.9%). Most of the subjects showed larger primitive tumor (maximum diameter >3 cm) (n=117, 79.6%). Moreover, there were no statistically significant differences between the two cohorts with respect to age, gender, smoking history, tumor size, and PD-L1 expression levels. However, it should be noted that the predominant histopathological subtype in the PT_deep_ cohort was adenocarcinoma (59.1%), whereas squamous cell carcinoma was the primary subtype observed in the PTsup cohort (68.0%) (**Table 2**). Regarding the concomitant drugs, 81.0% of the subjects received chemotherapy and 7.5% received anti-angiogenic therapy at the same time. In detail, 72.7% (n=32) of the patients in the PT_deep_ cohort and 84.5% (n=87) in the PT_sup_ cohort received chemotherapy, respectively. Meanwhile, 9.1% (n=4) of the patients in the PT_deep_ cohort and 6.8% (n=7) in the PT_sup_ cohort had anti-angiogenic therapy. There was no statistical significance between two cohorts.

**Table 1:** Demographic and clinical characteristics of all subjects.

Variables	No. of patients (n=147)
Age (years)	68.0 (63.0,73.0)
Gender	
Male	133 (90.5%)
Smoke history	
Yes	93 (63.3%)
Histology	
Squamous cell carcinoma	88 (59.9%)
Non-squamous cell carcinoma	59 (40.1%)
Measurable tumor maximum diameter	
≤3 cm	30 (20.4%)
>3 cm	117 (79.6%)
Concomitant therapy	
Chemotherapy	119 (81.0%)
Anti-angiogenic therapy	11 (7.5%)
PD-L1 level expression (22C3)	40% (4%, 70%)
<50%	80 (54.4%)
≥50%	67 (45.6%)

**Table 2:** Demographic and clinical characteristics of two cohorts.

Variables	No. of patients		P-value
	PT_sup_ (n=104)	PT_deep_ (n=43)	
Age (years)	68 (63.0, 75.0)	67.5 (59.0, 71.8)	0.731
Gender			
Male	94 (91.3%)	39 (88.6%)	0.619
Smoke history			
Yes	65 (63.1%)	28 (63.6%)	0.951
Histology			0.002
Squamous cell carcinoma	70 (68.0%)	18 (40.9%)	
Non-squamous cell carcinoma	33 (32.0%)	25 (59.1%)	
Maximum diameter			
>30 mm	81 (78.6%)	36 (81.8%)	0.662
PD-L1 TPS			0.732
<50%	57 (55.3%)	23 (52.3%)	
≥50%	46 (44.7%)	21 (47.7%)	
Concomitant therapy			
Chemotherapy	87 (84.5%)	32 (72.7%)	0.097
Antiangiogenic therapy	7 (6.8%)	4 (9.1%)	0.628

Bold values means P<0.05 and have statistical significance.

### 3.2 Overview of PD-L1 detection and ICB-based therapy efficacy

In the entire population, 54.4% of subjects were diagnosed with negative or low PD-L1 TPS (<50%), and 45.6% of subjects had high PD-L1 TPS (≥50%). As for the efficacy of ICB-based therapy, the DCR rate was 91.8% (135/147) and the ORR rate was 24.5% (36/147) in the entire population. More specifically, the tumor response of all subjects was PR in 36 patients, SD in 99 patients, and PD in 12 patients. Considering the relatively high DCR rate, our study mainly focused on the relationship between PD-L1 expression and ORR. Then, we performed logistic analysis of the relationship between random PD-L1 TPS and ORR after two cycles of ICB-based therapy. As shown in **Table 3**, we found that the high level of random PD-L1 TPS significantly correlated with higher ORR (OR 2.694, 95% CI 1.236-5.872, P = 0.013). Moreover, random PD-L1 TPS remained an independent predictive biomarker of ORR after correcting for potential confounding factors including tumor size and distant metastasis (OR 2.735, 95% CI 1.215-6.154, P = 0.015).

**Table 3:** Logistic regression analysis of ORR in all subjects.

Characteristics	Univariate analysis			Multivariate analysis		
	OR	95% CI	P	OR	95% CI	P
Age	0.984	0.946-1.024	0.429	0.979	0.936-1.024	0.349
Smoke	0.885	0.408-1.920	0.758	–	–	–
Histology	0.933	0.432-2.015	0.861	–	–	–
Maximum diameter	0.555	0.195-1.577	0.269	0.597	0.203-1.758	0.349
Distant metastasis	0.390	0.109-1.393	0.147	0.369	0.100-1.359	0.134
Chemotherapy	0.769	0.306-1.936	0.577	0.810	0.282-2.332	0.697
Random PD-L1 level	2.694	1.236-5.872	**0.013**	2.735	1.215-6.154	**0.015**

Bold values means P<0.05 and have statistical significance.

### 3.3 Weak ability of PD-L1 located in the superficial tumor subregion to predict the ORR of ICB

**Figure 3:**
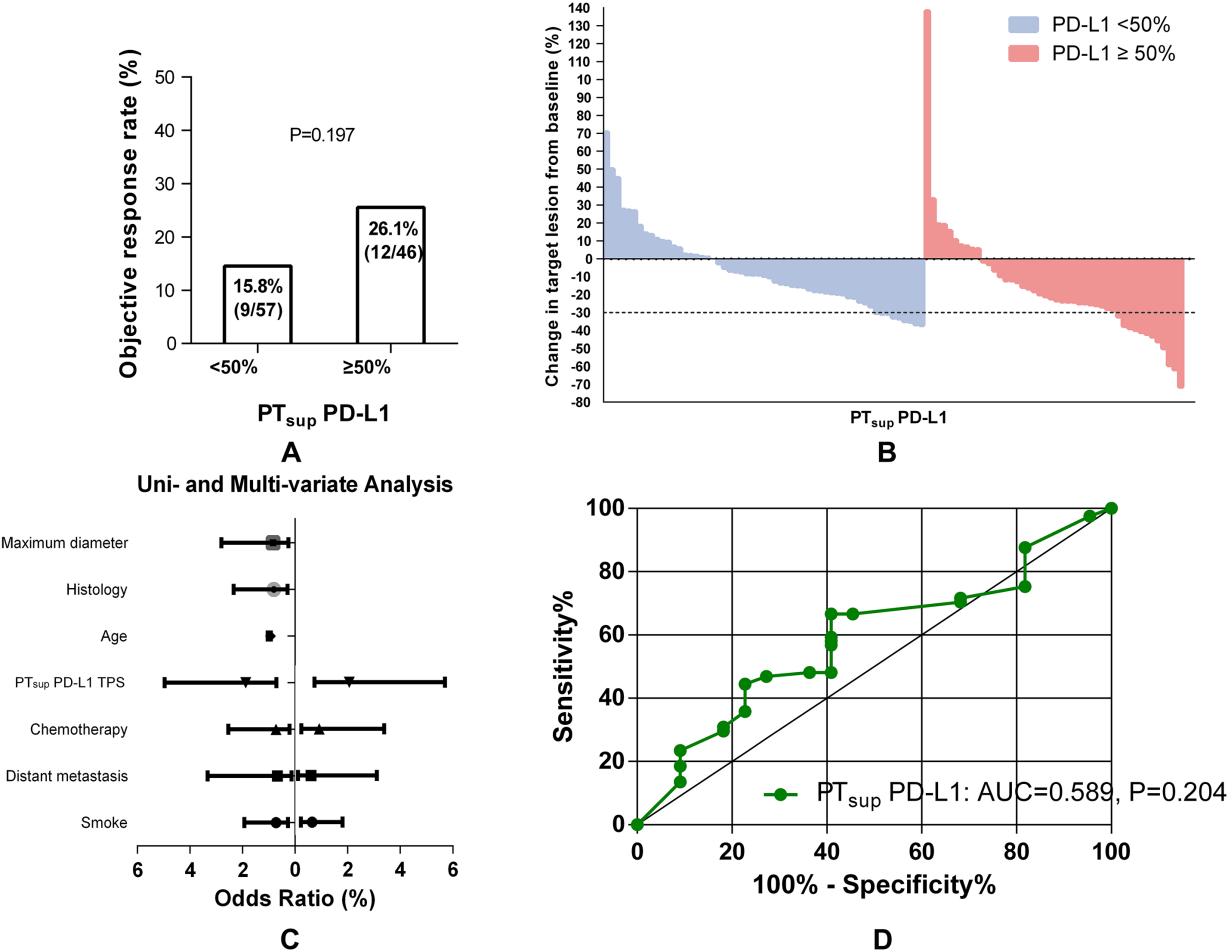
Association between PT_sup_-related PD-L1 TPS and ORR. Histograms showed the comparison of ORR in patients with PT_sup_-related PD-L1 TPS <50% or ≥50% **(A)**. The best objective response to ICB-based therapy is shown as a percent change of target lesions from baseline in evaluable patients in the PT_sup_ cohort **(B)**. The uni- and multivariate logistic analyses of ORR in the PT_sup_ cohort **(C)**. ROC curve analysis of the predictive value of PT_sup_-related PD-L1 TPS **(D)**.

To date, IHC-based detection of PD-L1 TPS is still problematic in determining ICB-based therapy effect accurately. One well-known issue was that spatial heterogeneity impacts the performance of PD-L1 as a biomarker for ICB efficacy. Then, we used a cohort of NSCLC patients sampled from the primary tumor superficial subregion to evaluate the predictive efficacy of PT_sup_-related PD-L1 TPS (**Figure 3**). Interestingly, PT_sup_-related PD-L1 TPS ≥50% was not associated with the better ORR under ICB-based therapy (15.8% (9/57) vs. 26.1% (12/46), P = 0.197, **Figure 3A**). Moreover, logistic analysis showed that PT_sup_-related PD-L1 TPS was not the independent predictive biomarker of ORR (OR 1.882, 95% CI 0.714-4.963, P = 0.201; OR 2.076, 95% CI 0.754-5.715, P = 0.157) (**Figure 3C**). To ensure the robustness of PT_sup_-related PD-L1 TPS as a continuous variable for ICB-based predictive efficacy, we further performed ROC curve analysis. As shown in **Figure 3D**, PT_sup_-related PD-L1 TPS had a relatively low AUC (AUC=0.589, P=0.204). Therefore, PT_sup_-related PD-L1 TPS was a weak biomarker to predict the ORR of ICB-based therapy.

### 3.4 Robustness of the predictive efficiency of PD-L1 located in the primary tumor deep subregion

**Figure 4:**
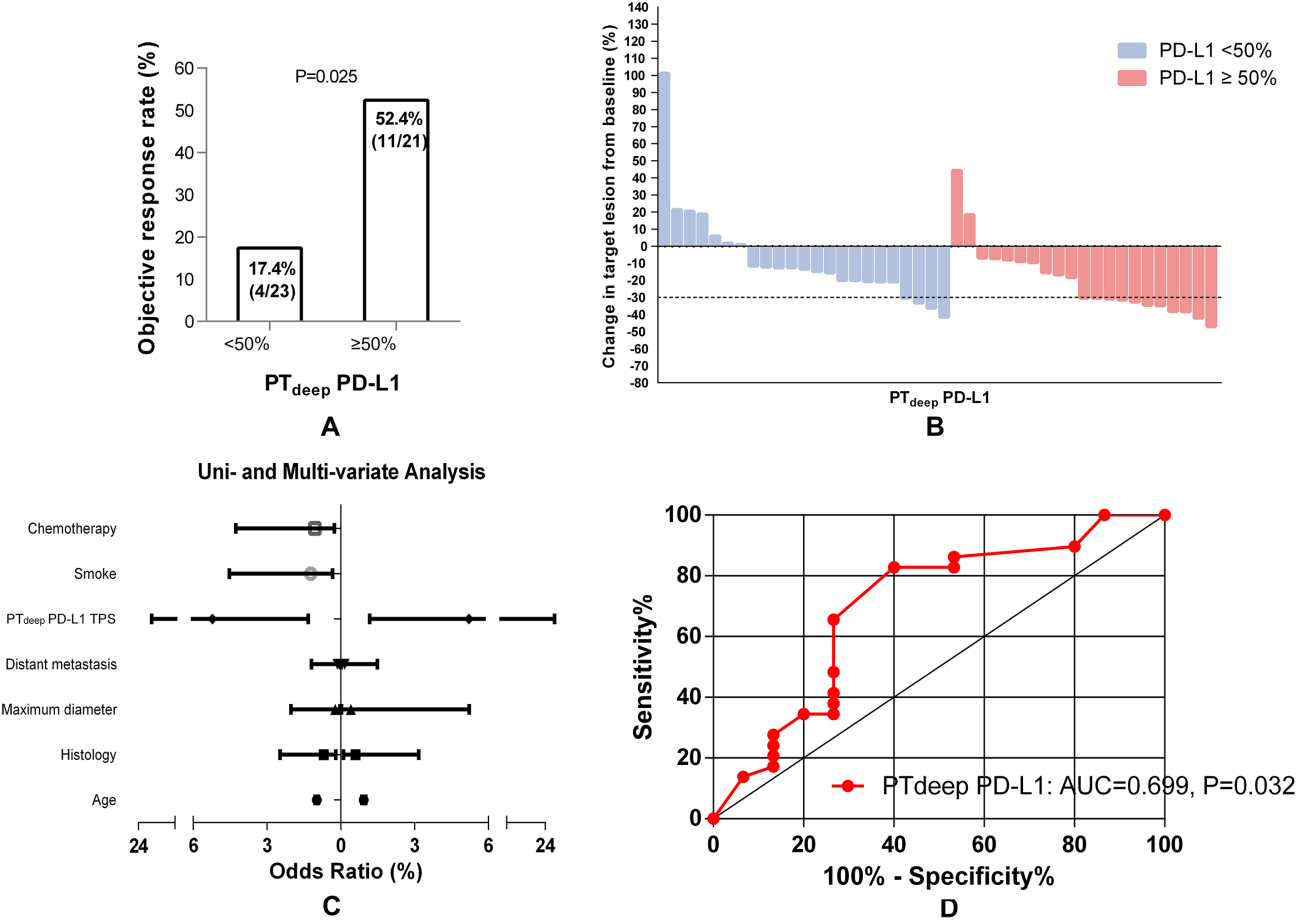
PT_deep_-related PD-L1 TPS correlates of the response to first-line ICB-based therapy in advanced NSCLC patients. Higher PT_deep_-related PD-L1 TPS was associated with a higher ORR of ICB-based therapy **(A)**. The best objective response to ICB-based therapy is shown as a percent change of target lesions from baseline in evaluable patients in the PT_deep_ cohort **(B)**. PT_deep_-related PD-L1 TPS was the independent predictor of ORR in ICB-based therapy **(C)**. ROC curve analysis of the predictive value of PT_deep_-related PD-L1 TPS **(D)**.

Inversely, NSCLC patients with PT_deep_-related PD-L1 TPS ≥50% could hold a substantially higher ORR than those with TPS <50% (52.4% (4/23) vs. 17.4% (11/21), *P* = 0.025) (**Figure 4A, B**). Considering the strong association of PT_deep_-related PD-L1 TPS with the ORR, we next looked to determine whether that was the robustly affected ORR from different dimensions. Univariable and multivariable logistic analyses (**Figure 4C**) confirmed again that it was still positively linked to the ORR by PT_deep_ PD-L1 TPS (OR 5.225, 95% CI 1.319-20.705, P = 0.019; OR 5.386, 95% CI 1.212-23.937, P = 0.027). Moreover, ROC curve analysis (**Figure 4D**) supplemented the conclusion that the predictive value of PT_deep_-related PD-L1 TPS was accurate (AUC = 0.699, P = 0.032). Overall, these data supported the presence of tumor heterogeneity between primary tumor superficial and deep subregions, especially in the association with the ORR of ICB-based therapy.

### 3.5 PT_deep_-related PD-L1 TPS ≥50%: a strongest biomarker to predict ORR

**Figure 5:**
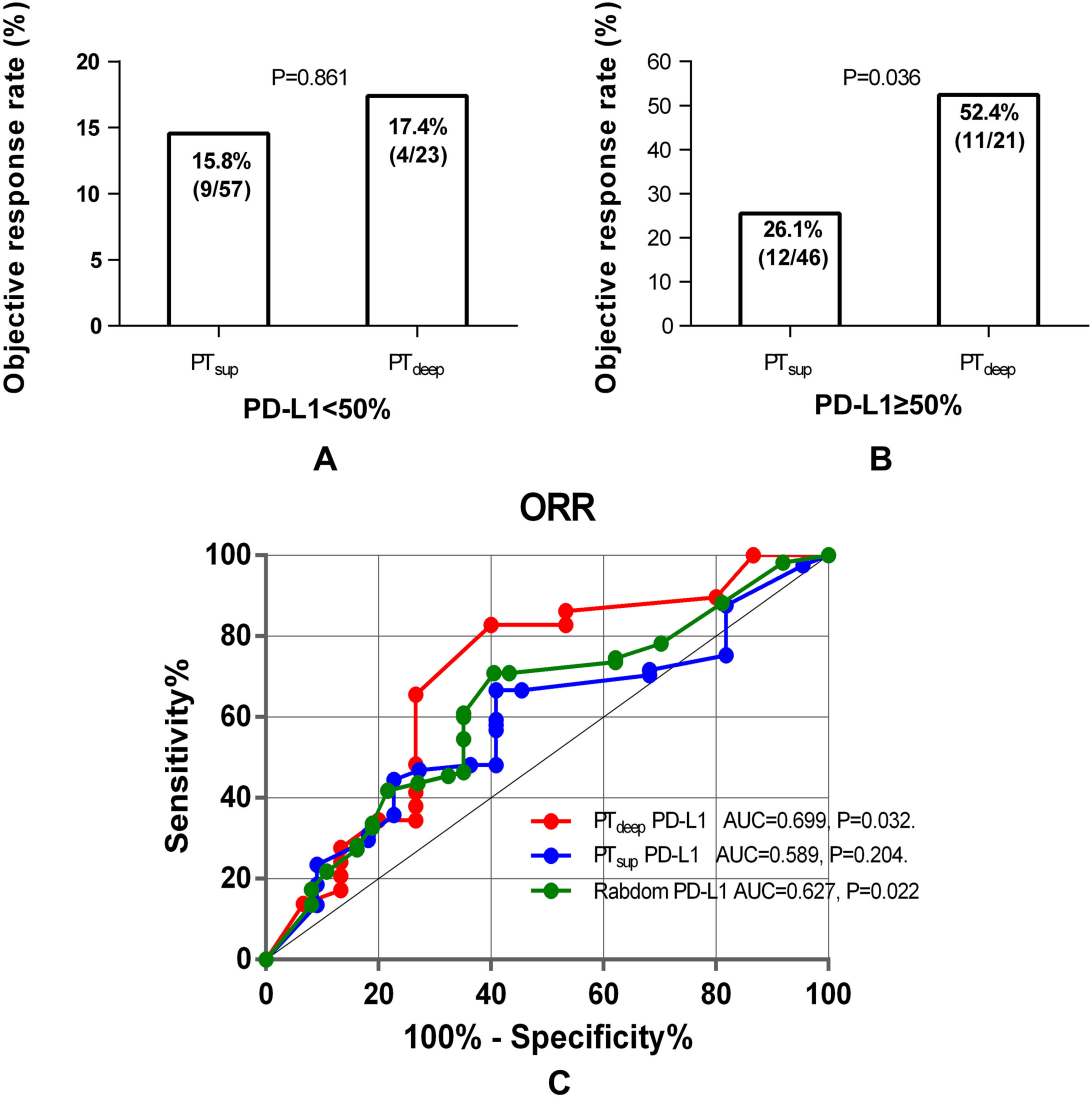
Cross analysis of PT_deep_- and PT_sup_-related PD-L1 TPS to predict ORR. Patients with PD-L1 TPS <50% in the PT_deep_- and PT_sup_ cohorts reached similarly low ORRs **(A)**. Patients with PT_deep_-related PD-L1 TPS ≥50% were associated with significantly higher ORRs than those with PD-L1 TPS ≥50% in the PT_sup_ cohort **(B)**. ROC curve analysis of PT_sup_, PT_deep_, and random PD-L1 TPS in predicting ORR **(C)**.

Furthermore, cross comparison analysis between PT_sup_ and PT_deep_ cohorts displayed that PD-L1 TPS <50% from the superficial subregion or deep subregion in primary tumor reached similarly low ORRs (15.8% (9/57) vs. 17.4% (4/23), P = 0.861) (**Figure 5A**). On the contrary, patients with PD-L1 TPS ≥50% in the PT_deep_ cohort had a significantly higher ORR than those with PD-L1 TPS≥50% in the PT_sup_ cohort (**Figure 5B**). Moreover, we assessed the predictive efficiency of PD-L1 TPS from the superficial subregion or deep subregion in primary tumor at the cutoff value of 50% by diagnosis analysis (**Table 4**). We also observed that PT_deep_-related PD-L1 TPS (at the cutoff of 50%) was associated with better sensitivity (73.3% vs. 57.1%), specificity (65.5% vs. 58.5%), accuracy (68.1% vs. 58.3%), and Youden index (0.39 vs. 0.16) than PT_sup_ PD-L1 TPS in predicting ORR.

**Table 4:** The diagnosis analysis of PT_deep_- and PT_sup_-related PD-L1 TPS at the cutoff value of 50% to predict ORR.

Items	PT_deep_	PT_sup_
Sensitivity (%)	73.3	57.1
Specificity (%)	65.5	58.5
Accuracy (%)	68.1	58.3
False negative rate (%)	26.7	42.9
False positive rate (%)	34.5	41.5
Positive predictive value (%)	52.4	26.1
Negative predictive value (%)	82.6	84.2
Positive likelihood ratio	2.13	1.38
Negative likelihood ratio	0.41	0.73
Youden index	0.39	0.16

Lastly, we enrolled random, PT_deep_-related, and PT_sup_-related PD-L1 TPS into ROC curve analysis and then compared it by assessing the predictive value for the ORR of these three methods. As indicated in **Figure 5C**, PT_deep_-related PD-L1 achieved better discriminative performance with an AUC of 0.699 (P = 0.032), which was higher than those of PT_sup_-related PD-L1 TPS (AUC=0.589, P=0.204) and random PD-L1 TPS (AUC=0.627, P = 0.022). Therefore, this finding further substantiated the different predictive values of PD-L1 molecules expressed in distinct tumor subregions, whereas PT_deep_-related PD-L1 demonstrated superior ability to predict the ORR.

### 3.6 Longer PFS benefited from PT_deep_-related PD-L1 TPS ≥50%

**Figure 6:**
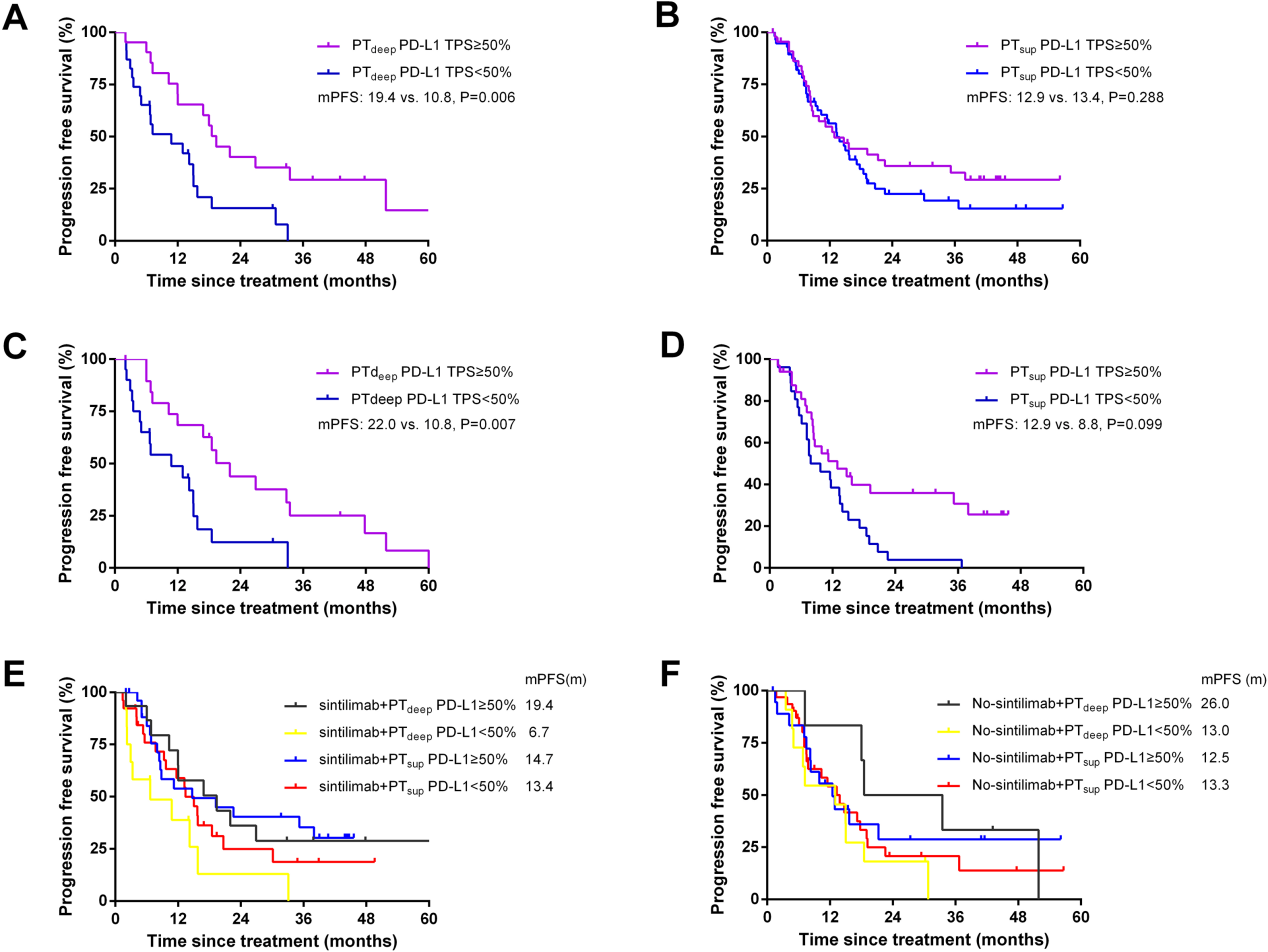
Better PFS benefited from PT_deep_-related PD-L1 TPS ≥50%. Patients with PT_deep_-related PD-L1 TPS ≥50% had a significantly superior PFS compared with patients with PT_deep_-related PD-L1 TPS <50% **(A)**. Patients in the PT_sup_ cohort showed similar mPFS in PD-L1 TPS ≥50% and <50% **(B)**. After matching gender and histology, patients with PT_deep_-related PD-L1 TPS ≥50% still achieved longer mPFS **(C)**. PFS were comparable between patients with PD-L1 TPS ≥50% and <50% in the PT_sup_ cohort **(D)**. Patients with PT_deep_-related PD-L1 TPS ≥50% had the longest mPFS in the sintilimab treatment subgroup **(E)**. Patients with PT_deep_-related PD-L1 TPS ≥50% had the longest mPFS in the no-sintilimab treatment subgroup **(F)**.

Considering that the short evaluation window may misclassify treatment benefit, we then expanded the prognostic value of PD-L1-TPS to PFS. In the PT_deep_ cohort, patients with PD-L1 TPS ≥50% had a significantly superior PFS (mPFS 19.4 vs. 10.8 months; P = 0.006) compared with patients with PD-L1 TPS <50% (shown in **Figure 6A**). Conversely, in the PT_sup_ cohort, PFS were comparable between patients with PD-L1 TPS ≥50% and <50% (mPFS:12.9 vs. 13.4 months; P>0.05; **Figure 6B**). This finding about the outstanding predictive value of PT_deep_-related PD-L1 TPS ≥50% to PFS was consistent with that to ORR.

### 3.7 Propensity score matching and stratified analysis

To minimize the potential impact of confounding factors, we performed 1:1 propensity score matching (PSM) analysis and stratified analyses. We mainly matched confounding factors including gender and histology, leading to the formation of 20 matched patient pairs in the PT_deep_ cohort and 33 matched patient pairs in the PT_sup_ cohort. As shown in **Figures 6C, D**, patients with PT_deep_-related PD-L1 TPS ≥50% still achieved longer mPFS (22.0 vs. 10.8 months, P = 0.006). Moreover, PT_sup_-related PD-L1 TPS still showed limited value to predict PFS (mPFS: 12.9 vs. 8.8 months, P>0.05). Therefore, these findings after matched analysis conformed to those of pre-PSM analysis.

To further survey the impact of the treatment heterogeneity on the relationship between subregional PD-L1 expressions and PFS benefit, stratified analysis was performed according to the application of different immunotherapy agents. The majority of patients enrolled in this study selected sintilimab as their immunotherapy agent (PT_deep_ cohort: 61.4%, n=27; PT_sup_ cohort: 51.5%, n=53). Based on this proportion, we then classified patients in two cohorts to sintilimab and no-sintilimab subgroups. It was found that patients with PT_deep_-related PD-L1 TPS ≥50% had longer PFS, irrespective of the specific immunotherapy agents employed (shown in **Figures 6E, F**). Altogether, these findings indicated that PT_deep_-related PD-L1 TPS ≥50% was the most stable biomarker to predict the short- and long-time efficacies of ICB-based treatment.

## 4 Discussion

PD-L1 remains the only approved biomarker by FDA for immune checkpoint blockade therapy with anti-PD-1 in advanced NSCLC. An appropriate treatment decision in NSCLC is highly correlated with the tumor PD-L1 expression level (19). The selected area for PD-L1 expression analysis, representing the tumor immunobiology environment, becomes a significant cause of spatial heterogeneity in PD-L1 expression.

PD-L1 expression has been widely studied since the early time of ICB therapy. At present, the majority of studies have suggested that NSCLC patients with a higher PD-L1 expression have a better therapeutic effect on receiving ICB therapy (5, 20). In detail, the ORR rate, progress-free survival, and overall survival of patients with a positive PD-L1 expression were significantly higher than those of patients with a negative PD-L1 expression (8, 21). In accordance with the previous studies, our study has suggested that random PD-L1 was the effective biomarker to predict the ORR of ICB-based therapy.

Notably, the results of most studies were more susceptible to the substantial intratumor heterogeneity due to the fact that they were mainly based on a single biopsy site, whereas others relied on archival tissue in which the PD-L1 expression might change over time (22). Recent studies have indicated that the classification of PD-L1 expression with small biopsy samples might not represent the overall expression of the PD-L1 level in considerable percentages of lung cancers (23). Moreover, the same trend was observed from another clinical study by Nir Hirshoren et al. and colleagues. One of their main findings was the CPS category of PD-L1 level inconsistency when comparing different areas within the same excised tumor. Moreover, this study demonstrated that the tumor leading-edge and near-dense inflammatory cell infiltration showed a higher CPS category, which could reflect the tumor immune-biology environment (13).

However, quite a few patients with NSCLC are just diagnosed at advanced stages only on the basis of small biopsy specimens in clinical practice because of the limitations of disease conditions. Therefore, optimizing the predictive value of PD-L1 in different small biopsy sites is relevant for clinical decision-making and for clinical trial design.

Considering the fact that the spatial heterogeneity may influence the tumor biopsy strategy and treatment planning (9), we should further explore the impact of spatial heterogeneity on the predictive value of PD-L1 expression on the ICB treatment in NSCLC patients. However, selection of an optimal site and modality for biopsy should be driven by the assessment of the clinical extent of disease in the lung, the intrathoracic lymph nodes, and the imaging (18). Therefore, all subjects in our study were further divided into tumor surface-sampled cohort (TBLB, transbronchial mucosal biopsy) and intratumor-sampled cohort (EBUS-TBNA, PCNB).

Our study has reported for the first time that the different biopsy sites (primary tumor deep or superficial subregion-sampled) of NSCLC impacted the performance of PD-L1 as a predictive biomarker for ICB-based therapy. In view of the results of logistic regression analysis and ROC curve analysis, PT_deep_-related PD-L1 TPS was undoubtedly a better biomarker to predict ORR from ICB-based therapy than PT_sup_-related PD-L1 TPS. Cross comparison analysis between PT_sup_ and PT_deep_ cohorts displayed that PD-L1 TPS <50% from the superficial subregion or deep subregion in primary tumor reached similarly low ORs. On the contrary, patients with PD-L1 TPS ≥50% in the PT_deep_ cohort had a significantly higher ORR than those with PD-L1 TPS ≥50% in the PT_sup_ cohort. Moreover, according to the diagnosis analysis (shown in **Table 4**), PT_deep_-related PD-L1 TPS performed more outstandingly than PT_sup_-related PD-L1 TPS, which could further prove the robustness of the predictive efficiency of PT_deep_-related PD-L1 TPS. In conclusion, the spatial distribution of PD-L1 in the primary tumor needs accurate assessment and PT_deep_-related PD-L1 TPS especially expressing ≥50% needs more attention.

Considering that the short evaluation window may misclassify treatment benefit, we then expanded the prognostic value of PD-L1-TPS to PFS. As shown in **Figure 6**, patients with PT_deep_-related PD-L1 TPS ≥50% still achieved longer mPFS than those with PT_deep_-related PD-L1 TPS <50%. Moreover, PT_sup_-related PD-L1 TPS still showed limited value to predict PF. Then, we performed PSM and stratified analyses to minimize the potential impact of confounding factors such as gender, histology, and immunotherapy agents. As expected, PT_deep_-related PD-L1 TPS still manifested outstanding value to predict PFS rather than PT_sup_-related PD-L1 TPS. Therefore, patients with PT_deep_-related PD-L1 TPS ≥50% had both higher ORR and longer PFS, which reminded that short-term favorable ORR may contribute to long-term stable disease control. On this basis, we concluded that PT_deep_-related PD-L1 TPS was the stronger and more stable biomarker to predict ORR and PFS.

In addition, different treatments could potentially influence the PD-L1 expression (24). Chemotherapy, radiotherapy, and targeted molecular therapy were reported to increase PD-L1 expression and upregulating PD-L1 is one approach cancer cells may apply to evade immune-mediated cell killing (25, 26). However, in this study, the PD-L1 expression testing was prior to all the relevant therapy to achieve the original PD-L1 expression. We are extremely grateful for pointing out the problem. Just as shown in **Figures 3C**, **4C**, the combination of chemotherapy was not associated with ORR after two cycles in both the PT_deep_ and PT_sup_ cohorts according to the logistic analysis. Additionally, we constructed a COX model as a supplementary analysis (shown in **Supplementary Table 1** and **Supplementary Table 2**). The results indicated that combination chemotherapy was not a prognostic factor for PFS in either the PT_deep_ or PT_sup_ group. Therefore, we suggest that combination chemotherapy may not provide significant improvement in tumor therapy. As for the limitations of our study, the deep subregional samples obtained via EBUS-TBNA or PCNB might not fully represent the intratumor heterogeneity of primary tumor, leading to an inaccurate assessment of PD-L1 expression. Therefore, the future studies could consider multiple sampling points within each deep subregion to improve representativeness. Moreover, samples are obtained by different biopsy methods from different patients; in fact, the samples from different biopsy methods/sites should be compared in one patient. Finally, RECIST may not precisely describe the full spectrum of response observed after a two-cycle ICB-based therapy because the existence of pseudo-progression could not be excluded. Therefore, these intriguing outcomes should be interpreted with caution, and further larger prospective studies are warranted to address these critical questions.

## Data Availability

The raw data supporting the conclusions of this article will be made available by the authors, without undue reservation.
