# Supplementary material for: Investigating subregional PD-L1 expression within primary tumors to predict clinical outcomes in advanced NSCLC patients who received ICB-based therapy
Source: Front Oncol. 2025 Oct 17;15:1497279. doi: 10.3389/fonc.2025.1497279 (PMC12575117; doi:10.3389/fonc.2025.1497279)
Supplement: Supplementary file 1 [file Table1.docx]

**Supplementary Table 1 COX proportional-hazards model of PFS in PT_deep_ cohort**

| Characteristics | Univariate analysis | | | Multivariate analysis | | |
| --- | --- | --- | --- | --- | --- | --- |
|  | HR | 95%CI | P | HR | 95%CI | P |
| Age | 1.011 | 0.976-1.048 | 0.533 | - | - | - |
| Smoke | 0.753 | 0.378-1.500 | 0.420 | 0.814 | 0.400-1.656 | 0.570 |
| Histology | 1.070 | 0.538-2.125 | 0.847 | - | - | - |
| Maximum diameter | 0.837 | 0.250-2.801 | 0.772 | - | - | - |
| Distant metastasis | 2.168 | 0.922-5.097 | 0.076 | 1.136 | 0.513-2.515 | 0.752 |
| Chemotherapy | 1.862 | 0.811-4.279 | 0.143 | 1.499 | 0.640-3.512 | 0.352 |
| PT_deep_ PD-L1 TPS | 0.423 | 0.251-0.714 | 0.001 | 0.398 | 0.190-0.833 | 0.014 |
