# Supplementary material for: Investigating subregional PD-L1 expression within primary tumors to predict clinical outcomes in advanced NSCLC patients who received ICB-based therapy
Source: Front Oncol. 2025 Oct 17;15:1497279. doi: 10.3389/fonc.2025.1497279 (PMC12575117; doi:10.3389/fonc.2025.1497279)
Supplement: Supplementary file 2 [file Table2.docx]

**Supplementary Table 2 COX proportional-hazards model of PFS in PT_sup_ cohort**

| Characteristics | Univariate analysis | | | Multivariate analysis | | |
| --- | --- | --- | --- | --- | --- | --- |
|  | HR | 95%CI | P | HR | 95%CI | P |
| Age | 1.025 | 0.997-1.055 | 0.082 | 1.023 | 0.988-1.059 | 0.198 |
| Smoke | 0.738 | 0.453-1.203 | 0.223 | 0.824 | 0.497-1.366 | 0.453 |
| Histology | 0.773 | 0.453-1.319 | 0.345 | 0.919 | 0.513-1.646 | 0.777 |
| Maximum diameter | 0.592 | 0.323-1.085 | 0.090 | 0.641 | 0.348-1.181 | 0.154 |
| Distant metastasis | 0.988 | 0.472-2.069 | 0.975 | - | - | - |
| Chemotherapy | 0.793 | 0.425-1.481 | 0.467 | 0.959 | 0.442-2.080 | 0.915 |
| PT_sup_ PD-L1 TPS | 0.769 | 0.472-1.251 | 0.290 | 0.780 | 0.449-1.356 | 0.379 |
